# Effects of anti-TNF-α treatment on lipid profile in rheumatic diseases: an analytical cohort study

**DOI:** 10.1186/s13075-016-1148-1

**Published:** 2016-11-10

**Authors:** Shadi Hassan, Uzi Milman, Joy Feld, Lihi Eder, Idit Lavi, Shai Cohen, Devy Zisman

**Affiliations:** 1 Department of Internal Medicine, Carmel Medical Center, Haifa, Israel; 2 Clinical Research Unit, Clalit Health Services, Haifa and Western Galilee District, Haifa, Israel; 3 The Ruth and Bruce Rappoport Faculty of Medicine, Technion, Israel Institute of Technology, Haifa, Israel; 4 Department of Rheumatology, Carmel Medical Center, Haifa, Israel; 5 Women’s College Research Institute, Women’s College Hospital, Toronto, ON Canada; 6 Department of Medicine, University of Toronto, Toronto, ON Canada; 7 Department of Community Medicine and Epidemiology, Carmel Medical Center, Haifa, Israel

**Keywords:** TNF-α inhibitors, Lipid profile, Rheumatoid arthritis, Ankylosing spondylitis, Psoriatic arthritis

## Abstract

**Background:**

The aim was to assess the influence of long-term treatment with tumor necrosis factor alpha (TNF-α) inhibitors on total cholesterol (TC), triglycerides (TG), low-density lipoprotein (LDL), high-density lipoprotein (HDL), and atherogenic index (AI) in rheumatoid arthritis (RA), psoriatic arthritis (PsA), and ankylosing spondylitis (AS) patients.

**Methods:**

A retrospective cohort study was conducted on RA, PsA, and AS patients treated with TNF-α inhibitors for at least 270 days between 2001 and 2011. Levels of TC, TG, LDL, and HDL and the AI were compared with baseline values at 0–6, 6–12, 12–18, and 18–24 months. Patients were further subdivided into three groups according to their HMG CoA reductase inhibitor (statin) treatment status in order to assess their effect on the results.

**Results:**

The records of 311 patients (152 RA, 90 PsA, and 69 AS) were reviewed. TC and TG increased following treatment with TNF-α inhibitors, from 180.85 ± 2.12 mg/dl and 116.00 ± 3.55 mg/dl at baseline to 188.12 ± 2.35 mg/dl (*p* = 0.02) and 132.02 ± 4.63 mg/dl at 0–6 months (*p* < 0.01), respectively, and to 184.88 ± 2.09 mg/dl (*p* = 0.02) and 129.36 ± 4.32 mg/dl at 18–24 months (*p* < 0.01), respectively. AI increased following treatment with TNF-α inhibitors, from –0.032 ± 0.017 at baseline to 0.004 ± 0.019 at 18–24 months (*p* < 0.01). LDL decreased significantly in patients who were treated with statins before and during the entire study period, from 119.97 ± 2.86 mg/dl at baseline to 104.02 ± 3.57 mg/dl at 18–24 months (*p* < 0.01), in contrast to an increase in LDL values in patients who did not receive statins during the study.

**Conclusions:**

TNF-α inhibitor treatment was associated with a significant increase in TC and TG levels and the AI. Adding statins to the treatment was associated with a significant decrease in LDL levels.

## Background

Atherosclerosis is a major cause of morbidity and mortality in systemic autoimmune diseases. Inflammation is central to the pathogenesis of atherosclerosis and is an important risk factor for vascular disease [1]. An increase in the atherogenic index (AI) is associated with an increase in the atherosclerosis process [2]. It is therefore important to treat cardiovascular risk factors, including dyslipidemia, in these patients.

The influence of tumor necrosis factor alpha (TNF-α) inhibitors on lipid profile [3–17] and AI [3, 5, 7–9, 11, 13, 14, 17] in patients with rheumatologic diseases has been examined in several studies (Table 1), with inconsistent results. In some studies TNF-α inhibitors promoted an atherogenic lipid profile, while in others there was no such effect. None of the studies assessed the time-dependent impact of TNF-α inhibitors on the lipid profile of ankylosing spondylitis (AS), psoriatic arthritis (PsA), and rheumatoid arthritis (RA) patients as a group.

**Table 1:** Literature on the influence of TNF-α inhibitors on lipid profile

Author	Publication year	Place	Follow up (weeks)	Patient number	Disease	Drug	Research type	TC^a^	TG^a^	LDL^a^	HDL^a^	AI^a^
Popa et al. [4]	2005	The Netherlands	2	33	RA	ADA	Prospective	↑	↔	↔	↑	
Vis et al. [6]	2005	The Netherlands	6	69	RA	INF	Prospective	↑			↑	
Seriolo et al. [8]	2006	Italy	24	34	RA	ETA, ADA, INF	Prospective	↑	↔		↑	↔
Allanore et al. [10]	2006	France	30	56	RA	INF	Prospective	↑		↑	↑	
Popa et al. [9]	2007	The Netherlands	52	55	RA	INF	Prospective	↑	↑	↔	↔	↑
Tam et al. [11]	2007	China	14	19	RA	INF	Prospective	↑	↑	↑	↑	↔
Peters et al. [13]	2007	The Netherlands	48	80	RA	INF	Prospective	↔	↔		↔	↔
Wijbrandts et al. [16]	2009	The Netherlands	52	50	RA	ADA	Prospective	↔	↔	↔	↔	
Mathieu et al. [7]	2010	France	14	34	AS	ETA, ADA, INF	Prospective	↑	↔	↔	↑	↔
Castro et al. [12]	2011	Brazil	3	15	PA	INF	Prospective	↔	↑	↔	↔	
Daïen et al. [3]	2012		2–52	13 articles	RA	ETA, ADA, INF	Systematic review, meta-analysis	↑	↑	↔	↑	↔
Curtis et al. [5]	2012	United States	Median > 2 years	289	RA	TNFi	Retrospective	↑	↑	↑	↑	↔
Di Minno et al. [14]	2014		2–52	1707	RA	TNFi	Meta-analysis	↑	↑	↔	↑	↔
				30 articles	PsA							
					AS							
Souto et al. [15]	2015		12–24	25 articles	RA	ADA, INF	Systematic review, meta-analysis	↔	↔	↔	↔	
Chen et al. [25]	2015	Taiwan	24	48	RA	ETA, ADA	Prospective	↔	↔	↔	↑	↔
Present study	2016	Israel	104	311	RA	TNFi	Retrospective	↑	↑	↔	↔	↑
					PsA							
					AS							

^a^Only significant changes (*p* ≤ 0.05) at the end of the follow-up period were included

*TNF-α* tumor necrosis factor alpha, *TC* total cholesterol, *TG* triglycerides, *LDL* low-density lipoprotein, *HDL* high-density lipoprotein, *AI*, atherogenic index, *RA* rheumatoid arthritis, *AS* ankylosing spondylitis, *PsA* psoriatic arthritis, *ADA* adalimumab, *INF* infliximab, *ETA* etanercept, *TNFi* TNF inhibitors

↑ Increased,  ↔ Unchanged

Our objective was to assess the influence of TNF-α inhibitors treatment on the lipid profile and the AI of patients with AS, PsA, and RA at various time points up to 2 years of treatment.

## Methods

### Study population

A retrospective cohort analysis was conducted on the database of Clalit Health Services (CHS) in Haifa and Western Galilee districts in northern Israel. CHS is the biggest healthcare provider in Israel, with over 1 million members in this area (approximately 50 % of the total population of the region). CHS maintains a comprehensive computerized database with continuous input from pharmaceutical, medical, laboratory, and administrative computerized operators. The CHS database and our study cohort were described in a previous study [18]. Briefly, the database for biological agents included in the Israeli ‘health basket’ contains diagnoses of specific rheumatic diseases as determined by a rheumatologist. The data are linked through a unique national identification number to the pharmaceutical, medical, and laboratory databases.

**Fig. 1:**
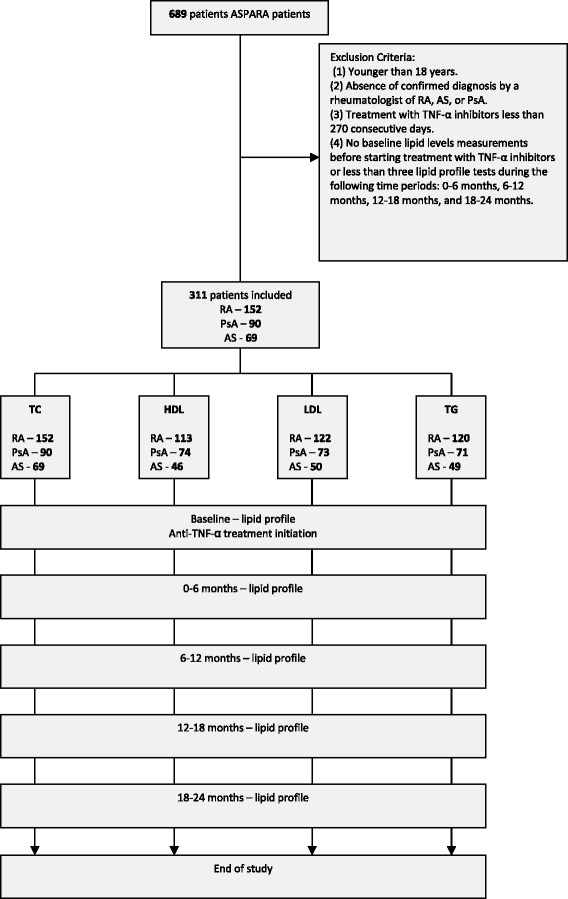
Study flow. *TNF-α* tumor necrosis factor alpha, *TC* total cholesterol, *TG* triglycerides, *LDL* low-density lipoprotein, *HDL* high-density lipoprotein, *RA* rheumatoid arthritis, *AS* ankylosing spondylitis, *PsA* psoriatic arthritis

Medical charts of patients who met the following criteria were reviewed: minimum 18 years old; diagnosis under one of the codes—‘rheumatoid arthritis’, ‘psoriatic arthritis’, and ‘ankylosing spondylitis’—and approval for biologic treatment included in the Israeli health basket; treated with TNF-α inhibitors between 2001 and 2011; began TNF-α inhibitors during the study period and were treated for at least 270 consecutive days; and had baseline lipid levels measured before starting treatment with TNF-α inhibitors and at least three lipid profile tests during the four time periods (0–6 months, 6–12 months, 12–18 months, and 18–24 months) (Fig. 1).

The following data were collected: demographics (age, gender); rheumatologist diagnosis (RA, PsA, AS); comorbidities (diabetes, hypertension, hyperlipidemia, ischemic heart disease); type and dates of pharmacy-dispensed medication (TNF-α inhibitors, steroids, disease-modifying anti-rheumatic drugs (DMARDs), HMG CoA reductase inhibitors (statins), fibrates); diabetes treatment; and laboratory tests results—lipid profile that included total cholesterol (TC), triglycerides (TG), low-density lipoprotein (LDL), and high-density lipoprotein (HDL). AI was calculated by the following formula: AI = log (TG/HDL), with TG and HDL expressed in molar concentrations [2].

The patients were subdivided into three groups according to statin treatment:

1. Patients not treated with statins.
2. Patients who started statin therapy during the study period, after the initiation of treatment with TNF-α inhibitors.
3. Patients who were treated with statins prior to and during the entire study period.

Patients from Groups 2 and 3 were included only if the type and dose of statin did not change during the study period. Patients treated with fibrates, which are known to reduce TG levels [19], were excluded from the analysis in the TG group.

### Statistical methods

Descriptive statistics are presented with continuous variables expressed as mean or median and standard deviation or standard error and categorical variables as frequencies and proportions. Comparisons of continuous patient characteristics among the three diagnostic groups (RA, PsA, AS) were performed by analysis of variance (ANOVA) or Kruskal–Wallis test, according to data distribution. Categorical variables were compared using the chi-square test.

The effect of TNF-α inhibitors therapy on lipid profile was assessed by comparing the levels of lipid particles at each time point with the baseline prior to treatment. Each lipid particle was analyzed in univariable and multivariable models, adjusted for the following study parameters: hyperlipidemia, statin treatment, steroid treatment, rheumatic diseases (RA, PsA, AS), obesity, smoking, diabetes, hypertension, ischemic heart disease, gender, and age.

Comparisons at various times were performed by repeated-measures mixed-model ANOVA. This procedure takes into account the intracorrelation of repeated measurements carried out on the same subject and does not exclude subjects with incomplete data at follow-up. Stratification analysis by statin use was employed in a multivariable model in patients treated concomitantly with a TNF-α inhibitor and statins after adjustment for hyperlipidemia, hypertension, obesity, gender, and age.

The analyses were conducted with the PASW (SPSS) 19 statistical package (PASW Software Statistic, SPSS, Chicago, IL, USA). All statistical tests were two-sided and statistical significance was considered *p* ≤ 0.05.

## Results

### Study population

Of the 689 patients treated with TNF-α inhibitors between 2001 and 2011, 311 met the inclusion criteria: 152 RA patients, 90 PsA patients, and 69 AS patients. The mean age of RA and PsA patients was higher (47.6 ± 19.3 and 50.2 ± 13.4 years, respectively) than that of AS patients (39.7 ± 15.2 years, *p* <0. 01). As expected, there were more females in the RA group (74.3 %) than in the AS group (36.0 %, *p* < 0.01). Corticosteroids were used more often in the RA group (58.7 %) than in the PsA and AS groups (29.2 % and 26.6 %, respectively, *p* < 0.01). Diabetes, hyperlipidemia, and hypertension were more prevalent in the PsA group (19.0 %, *p* = 0.03; 52.2 %, *p* = 0.02; and 40.0 %, *p* = 0.02, respectively) than in the RA group (11.2 %, 37.5 %, and 36.2 %, respectively) and the AS group (1.4 %, 32.0 %, and 20.3 %, respectively). The incidence of ischemic heart disease did not differ significantly between the groups. Smoking was more prevalent in the AS group (45.0 %, *p* = 0.02) than in the RA and PsA groups (26.3 % and 30.0 %, respectively). Baseline values of TC and LDL did not differ significantly between the groups. HDL and TG levels were significantly lower in patients with AS. Higher TG levels were found in PsA patients. There was no difference at baseline between the three groups in the percentage of patients treated with statins (Table 2). We excluded nine patients treated with fibrates from the TG group. These nine patients were treated with a constant dose of fibrates throughout the study and exhibited no significant changes in HDL and LDL values when TNF-α inhibitor was introduced; they were included in the TC, HDL, and LDL analysis.

**Table 2:** Baseline characteristics of patients with rheumatoid arthritis, psoriatic arthritis, and ankylosing spondylitis

		Rheumatoid arthritis	Psoriatic arthritis	Ankylosing spondylitis	*p* value
		(*n* = 152)	(*n* = 90)	(*n* = 69)	
Age (years)		47.6 ± 19.3	50.2 ± 13.4	39.7 ± 15.2	<0.0001
		50.0	52.0	38	
Baseline (mg/dl)	TC^a,b^	180.6 ± 37.6	186.4 ± 35.2	174.3 ± 38.9	NS
		183.0	185.0	172.0	
	HDL^a,c^	53.92 ± 15.67	51.23 ± 14.51	46.40 ± 13.31	0.003
		52.08	48.25	43.38	
	LDL^a,d^	108.77 ± 25.63	114.43 ± 22.31	111.67 ± 34.09	NS
		110.70	117.14	109.39	
	TG^a,e^	111.35 ± 53.44	128.36 ± 57.32	109.49 ± 53.87	0.04
		96.46	119.00	106.00	
Gender	Male	39 (25.7)	26 (28.9)	44 (64.0)	<0.0001
	Female	113 (74.3)	64 (71.1)	25 (36.0)	
Statin treatment	Treated before and during treatment with TNF-α inhibitors	25 (18.4)	22 (24.4)	9 (13.0)	NS
	Began treatment after beginning TNF-α inhibitors treatment	18 (11.8)	11 (12.2)	8 (11.6)	
Medication	Classical DMARDs^f^	93 (62.0)	35 (39.3)	20 (31.3)	<0.0001
	Steroids	88 (58.7)	26 (29.2)	17 (26.6)	<0.0001
Smoking	Current or past	40 (26.3)	27 (30.0)	31 (44.9)	0.02
Obesity		27 (17.8)	22 (24.4)	6 (8.7)	0.04
Hyperlipidemia		57 (37.5)	47 (52.2)	22 (32.0)	0.02
Diabetes		17 (11.2)	17 (18.9)	1 (1.4)	0.003
Hypertension		55 (36.2)	36 (40.0)	14 (20.3)	0.02
Ischemic heart disease		21 (13.8)	7 (7.8)	4 (5.8)	NS

Data presented as standard deviation with median below, or as *n* (%)

^a^Because of missing values of lipid profile at various time points, the number of patients for each lipid profile test differed

^b^TC: 152 RA, 90 PsA, and 69 AS patients

^c^HDL: 113 RA, 74 PsA, and 46 AS patients

^d^LDL: 122RA, 73 PsA, and 50 AS patients

^e^TG: 120 RA, 71 PsA, and 49 AS patients

^f^Classical DMARDs: leflunomide, azathioprine, cyclosporin, mercaptopurine, methotrexate, hydroxychloroquine, sulfasalazine

*TC* total cholesterol, *HDL* high-density lipoprotein, *LDL* low-density lipoprotein, *TG*, triglycerides, *TNF-α* tumor necrosis factor alpha, *DMARD* disease-modifying anti-rheumatic drug, *RA* rheumatoid arthritis, *AS* ankylosing spondylitis, *PsA* psoriatic arthritis, *NS* not significant

### Lipid profile

TC and TG levels increased following TNF-α inhibitor treatment, mainly during the first 6 months of treatment, and decreased thereafter but remained significantly higher than baseline at last follow-up (Table 3). LDL and HDL levels did not change significantly following introduction of TNF-α inhibitors.

**Table 3:** Effects of TNF-α inhibitor treatment on lipid profile

		Time				
		Baseline value before treatment	0–6 months	6–12 months	12–18 months	18–24 months
Total cholesterol (mg/dl)	Mean ± standard error	180.85 ± 2.12	188.12 ± 2.35	183.80 ± 2.14	184.48 ± 2.08	184.88 ± 2.09
	*p* value		<0.0001	NS	0.030	0.015
	Multivariate model^a^ *p* value		0.018	NS	0.041	0.018
HDL (mg/dl)	Mean ± standard error	51.8 ± 1.07	53.55 ± 1.21	51.06 ± 1.13	52.04 ± 1.18	52.05 ± 1.18
	*p* value		NS	NS	NS	NS
	Multivariate model^a^ *p* value		NS	NS	NS	NS
LDL (mg/dl)	Mean ± standard error	108.70 ± 1.55	109.00 ± 1.92	108.42 ± 1.76	108.54 ± 1.75	108.67 ± 1.70
	*p* value		NS	NS	NS	NS
	Multivariate model^a^ *p* value		NS	NS	NS	NS
Triglycerides (mg/dl)	Mean ± standard error	116.00 ± 3.55	132.02 ± 4.63	124.47 ± 3.95	128.72 ± 4.17	129.36 ± 4.32
	*p* value		<0.0001	0.005	<0.0001	<0.0001
	Multivariate model^a^ *p* value		<0.0001	0.004	<0.0001	<0.0001
Atherogenic index (LogTG/HDL)	Mean ± standard error	–0.032 ± 0.017	–0.001 ± 0.02	–0.01 ± 0.019	0.009 ± 0.018	0.004 ± 0.019
	*p* value		0.005	0.043	0.001	0.003
	Multivariate model^a^ *p* value		0.007	0.04	0.001	0.004

^a^Adjusted for hyperlipidemia, statin treatment, steroid treatment, rheumatic diseases (RA, AS, PsA), obesity, smoking, diabetes, hypertension, ischemic heart disease, gender, and age

*TNF-α* tumor necrosis factor alpha, *HDL* high-density lipoprotein, *LDL* low-density lipoprotein, *TG*, triglycerides, *NS* not significant, *RA* rheumatoid arthritis, *AS* ankylosing spondylitis, *PsA* psoriatic arthritis

The same pattern was found in the multivariate model after adjustment for hyperlipidemia, statin treatment, steroid treatment, rheumatic diseases (RA, PsA, AS), obesity, smoking, diabetes, hypertension, ischemic heart disease, gender, and age.

### Atherogenic index

The AI increased significantly following TNF-α inhibitor treatment (Table 3). The same increase was found in the multivariate model after adjustment for hyperlipidemia, statin treatment, steroid treatment, rheumatic diseases (RA, PsA, AS), obesity, smoking, diabetes, hypertension, ischemic heart disease, gender, and age.

### Co-administration of TNF-α inhibitors and statins

The effect of the interaction between TNF-α inhibitors and statins on the various lipid parameters after adjustment for hyperlipidemia, hypertension, obesity, gender, and age reached statistical significance for LDL (*p* < 0.01), TC (*p* = 0.02), and TG (*p* < 0.01) values (Table 4). No effect of the interaction was found in HDL values and therefore data were not analyzed further.

**Table 4:** Effects of TNF-α inhibitor treatment on lipid profile: stratification analysis by statin use

		No statins		Statin treatment begun after initiating TNF-α inhibitor treatment		Statin treatment begun before and continued during TNF-α inhibitor treatment	
	Time period	Mean ± standard error	*p* value	Mean ± standard error	*p* value	Mean ± standard error	*p* value
Total cholesterol^a^ (mg/dl)	Reference	183.73 ± 3.26		188.96 ± 8.92		182.04 ± 5.65	
	0–6 months	188.63 ± 3.42	0.007	208.22 ± 9.02	0.005	188.78 ± 5.73	NS
	6–12 months	185.20 ± 3.29	NS	197.92 ± 8.74	NS	184.05 ± 5.60	NS
	12–18 months	187.77 ± 3.22	0.03	198.55 ± 9.15	0.04	180.42 ± 5.48	NS
	18–24 months	188.62 ± 3.34	NS	199.38 ± 8.46	0.01	178.89 ± 5.14	NS
LDL^a^ (mg/dl)	Reference	103.13 ± 3.68		112.10 ± 5.62		119.97 ± 2.86	
	0–6 months	106.01 ± 3.83	0.057	123.42 ± 7.62	0.05	107.84 ± 3.96	<0.001
	6–12 months	105.63 ± 3.80	NS	115.35 ± 7.05	NS	108.26 ± 4.07	<0.001
	12–18 months	106.85 ± 3.80	0.02	119.67 ± 6.38	0.03	101.85 ± 3.57	<0.001
	18–24 months	107.70 ± 3.76	0.001	115.89 ± 6.81	NS	104.02 ± 3.57	<0.001
Triglycerides^a^ (mg/dl)	Reference	119.41 ± 9.75		179.31 ± 19.96		142.14 ± 9.89	
	0–6 months	134.33 ± 10.24	<0.001	223.91 ± 24.84	0.002	147.33 ± 10.67	NS
	6–12 months	128.80 ± 10.05	0.02	186.09 ± 20.47	NS	145.81 ± 10.15	NS
	12–18 months	135.89 ± 10.19	0.001	181.31 ± 20.98	NS	149.60 ± 10.16	NS
	18–24 months	135.99 ± 10.20	<0.001	194.68 ± 23.42	NS	146.72 ± 10.82	NS

^a^Adjusted for hyperlipidemia, hypertension, obesity, gender, and age

*TNF-α* tumor necrosis factor alpha, *LDL* low-density lipoprotein, *NS* not significant

LDL levels rose higher than baseline values (103.13 ± 3.68 mg/dl) in patients treated with TNF-α inhibitors but not with statins, reaching statistical significance at 12–18 months (106.85 ± 3.80 mg/dl, *p* = 0.024) and 18–24 months (107.70 ± 3.76 mg/dl, *p* = 0.001). LDL in patients who began statins after the initiation of TNF-α inhibitors reached peak value at 6 months and 12–18 months. In contrast, LDL decreased significantly in patients who were treated with statins before starting TNF-α inhibitors and continued throughout the study: from 119.97 ± 2.86 mg/dl at baseline to 107.84 ± 3.96 mg/dl at 0–6 months (*p* < 0.01), 108.26 ± 4.07 mg/dl at 6–12 months (*p* < 0.01), 101.85 ± 3.57 mg/dl at 12–18 months (*p* < 0.01), and 104.02 ± 3.57 mg/dl at 18–24 months (*p* < 0.01) (Table 4).

TC increased in patients not treated with TNF-α inhibitors or statins, from 183.73 ± 3.26 mg/dl at baseline to 188.63 ± 3.42 mg/dl at 0–6 months (*p* < 0.01) and 187.77 ± 3.22 mg/dl at 12–18 months (*p* = 0.03). In patients who began statins after the initiation of TNF-α inhibitors, TC increased from 188.96 ± 8.92 mg/dl at baseline to 208.22 ± 9.02 mg/dl at 0–6 months (*p* < 0.01), 198.55 ± 9.15 mg/dl at 12–18 months (*p* = 0.04), and 199.38 ± 8.46 mg/dl at 18–24 months (*p* = 0.01). TC values in patients treated with statins before initiation of TNF-α inhibitors did not change significantly.

The same pattern was observed in TG values, which increased significantly in patients treated with TNF-α inhibitors but not statins, and in patients who began statins after the initiation of TNF-α inhibitors, but only in the first 6 months. TG values increased, but not significantly, in patients treated with statins before TNF-α inhibitor initiation and during the whole study period (Table 4).

## Discussion

The demographic and clinical characteristics of our cohort are consistent with those described in the literature. AS patients were young and most were males, while RA patients were older, needed more treatment with corticosteroids, and most were females [20, 21]. A large meta-analysis by Armstrong et al. in 2013 [22] showed an increased risk of metabolic syndrome in patients with psoriasis (odds ratio 2.26). According to our data, diabetes, hyperlipidemia, and hypertension were more prevalent in PsA patients than in RA and AS patients (Table 2).

TNF-α inhibitors revolutionized the treatment of inflammatory arthritic conditions [23, 24]. In our study, TNF-α inhibitors significantly increased the AI, TC, and TG levels without significantly changing LDL and HDL levels. They also significantly increased LDL, TC, and TG levels in patients who were not treated with statins during the study. This was in contrast to the decrease in LDL levels in patients who were treated with statins prior to initiation of TNF-α inhibitors and continued treatment throughout the study.

A comparison of our results with those reported in the literature must take into account the different study designs. The 15 studies that assessed the effects of TNF-α inhibitors on lipid profile (Table 1) [3–17] were of shorter duration, ranging from 2 to 30 weeks in nine studies [4, 6–8, 10–12, 15, 17] and up to 52 weeks in six other studies [3, 5, 9, 13, 14, 16]. Follow-up in our study was 104 weeks. We conducted our analysis on pooled data of rheumatic patients in the ASPARA cohort while most of the published studies analyzed a single disease: 12 studies of RA, one study of PsA, and one study of AS. Sample size also varied: 15–80 patients in published studies [4, 6–13, 16, 17] compared with 311 patients in our cohort. Six of the studies were performed in patients treated with infliximab (INF) alone, and the rest included data on different types of TNF-α inhibitors such as INF, etanercept (ETA), and/or adalimumab (ADA) (11 of 15 studies) [3, 4, 6–13, 15, 17]. Our study was performed in patients treated with INF, ETA, or ADA.

Our finding of a significant increase in TC levels following TNF-α inhibitor treatment (Table 3) is in line with the results of 10 of the 15 studies mentioned [3–11, 14]. Six of the 15 studies also reported an increase in TG levels following TNF-α inhibitors [3, 5, 9, 11, 12, 14], similar to our findings, while the other nine showed no significant changes in TG levels. Nine of the 15 studies studied the effect of TNF-α inhibitors on the AI [3, 5, 7–9, 11, 13, 14, 17]. Only one of them showed an increase in the AI [9], similar to our results, while the others did not demonstrate significant changes of the AI. Concomitant treatment with statins, known to reduce TG, TC, LDL, and TNF-α levels [25, 26], could account for the lack of increase in TG levels and consequently the AI in those studies. Three studies that showed an increase in TG values included patients on drugs that affect the lipid profile, such as statins [5, 9, 12]; the studies that did not show significant changes in TG levels did not include them in their calculations [4, 6–8, 10, 13, 15, 16].

Our results showed no significant changes in the HDL and LDL values following TNF-α inhibitor treatment. The 10 studies from the literature [3–8, 10, 11, 14, 17] (Table 1) that did report increased levels of HDL could be explained by the longer follow-up period in our study. Wijbrandts et al. [16] reported a significant increase in HDL levels after 16 weeks of ADA treatment, although the level returned to baseline by the end of the 52-week study. Similarly, HDL levels in our study increased during the first 24 weeks and returned to near baseline levels later, although none of these changes was statistically significant (Table 3). Like in our cohort, nine of the 15 studies that assessed the effects of TNF-α inhibitors on lipid profile [3, 4, 7, 9, 12, 14–17] showed no significant change in LDL level following TNF-α inhibitor treatment. Because this could be due to concomitant treatment with statins, which can lower LDL values [25, 26], we tested whether there was an interaction between TNF-α inhibitor and statin treatment that affected the lipid profile.

LDL, TG, and TC levels increased significantly in patients treated with TNF-α inhibitors but not with statins. LDL and TC levels also increased significantly in patients who began statin therapy during the study, while TG increased significantly only during the first 6 months in this group. LDL values in patients treated with statins before and during the entire study period decreased significantly throughout the study. Thus, concomitant treatment with TNF-α inhibitor and statins may protect against the expected increase in LDL, helping reduce the cardiovascular risk in rheumatic patients in addition to treating the inflammatory component.

To our knowledge, no other study has demonstrated the benefit to LDL levels of adding statin treatment to TNF-α inhibitors. A meta-analysis of 31 observational studies showed that the TNF-α gene is involved in the pathogenesis of the metabolic syndrome (the *308A* variant of this gene has been associated with elevated levels of insulin and blood pressure) [27]. Another study reported an association between polymorphisms of TNF-α (TNF-αG-238A) and LDL level [28]. These investigators also found that *TNF-α-C-857T*—a polymorphism of the TNF-α gene promoter that increases transcription—is associated with increased LDL values and the formation of atherosclerotic carotid plaques in type 2 diabetes patients. Carriers of *TNF-α-C-857T* had significantly increased LDL values compared with noncarriers. There was no difference in LDL values in patients who were not treated with statins regardless of the carrier state of *TNF-α-C-857T*. In contrast, carriers of *TNF-α-C-857T* who were treated with statins had increased LDL levels compared with noncarriers [29]. In other words, there is a link between statin therapy, TNF-α activity level, and LDL. These findings, together with our results, call for further research into the impact on LDL levels of adding statin treatment in patients with increased activity levels of TNF-α (as in carriers who harbor the *TNF-α-C-857T* polymorphism) compared with patients with low activity levels of TNF-α (such as those receiving TNF-α inhibitors).

There are some limitations in our study. In addition to being a retrospective and observational study without a control group, the study was conducted on a relatively small cohort of patients which included those who were taking various medications, including steroids and fibrates, and had illnesses such as diabetes that could affect lipid metabolism. We dealt with this limitation by constructing statistical models for each lipid profile parameter adjusted for possible confounders (Tables 3 and 4). Being a retrospective study, follow-up data were not complete on all subjects; nevertheless, in order to include them in the analyses, we used repeated measures with mixed models that allow for unbalanced data and calculated the intracorrelation of repeated measurements carried out on the same subject. We estimated the drug intake according to pharma dispensed prescriptions. It would be interesting to evaluate the effects of long-term anti-TNF therapy on carotid artery US findings and its association with lipid profiles changes in further studies.

## Conclusions

The study documents the real-life, long-term follow-up and treatment of RA, PsA, and AS patients being treated with TNF-α inhibitors. We found that this treatment promotes an atherogenic lipid profile most pronounced in patients not treated by statins, evidenced by a significant increase in the AI, LDL, TC, and TG levels. Statin therapy before and during treatment with TNF-α inhibitors may protect against the expected rise in LDL values. While there were negative changes in the lipid profile under TNF-α inhibitor treatment, they were relatively small and not statistically significant, and their clinical significance is unclear.

Coronary disease is the main cause of death in rheumatic diseases and the importance of reducing cardiovascular risk factors, including dyslipidemia, in these patients cannot be overemphasized. Prospective studies on the long-term effects of TNF-α inhibitors on lipid profile, with and without statins, are required to assess the cardiovascular risk in these patients.

## Abbreviations

ADA: Adalimumab
AI: Atherogenic index
AS: Ankylosing spondylitis
DMARD: Disease-modifying anti-rheumatic drug
ETA: Etanercept
HDL: High-density lipoprotein
INF: Infliximab
LDL: Low-density lipoprotein
PsA: Psoriatic arthritis
RA: Rheumatoid arthritis
TC: Total cholesterol
TG: Triglycerides
TNF-α: Tumor necrosis factor alpha

## References

[1] Abou-Raya A, Abou-Raya S (2006). Inflammation: a pivotal link between autoimmune diseases and atherosclerosis. Autoimmun Rev.

[2] Shen S, Lu Y, Qi H, Li F, Shen Z, Wu L, Yang C, Wang L, Shui K, Wang Y, Qiang D, Yun J, Weng X (2016). Association between ideal cardiovascular health and the atherogenic index of plasma. Med (Baltimore).

[3] Daïen CI, Duny Y, Barnetche T, Daurès JP, Combe B, Morel J (2012). Effect of TNF inhibitors on lipid profile in rheumatoid arthritis: a systematic review with meta-analysis. Ann Rheum Dis.

[4] Popa C, Netea MG, Radstake T, Van der Meer JW, Stalenhoef AF, van Riel PL, Barerra P (2005). Influence of anti-tumour necrosis factor therapy on cardiovascular risk factors in patients with active rheumatoid arthritis. Ann Rheum Dis.

[5] Curtis JR, John A, Baser O (2012). Dyslipidemia and changes in lipid profiles associated with rheumatoid arthritis and initiation of anti-tumor necrosis factor therapy. Arthritis Care Res (Hoboken).

[6] Vis M, Nurmohamed MT, Wolbink G, Voskuyl AE, de Koning M, van de Stadt R, Twisk JW, Dijkmans BA, Lems WF (2005). Short term effects of infliximab on the lipid profile in patients with rheumatoid arthritis. J Rheumatol.

[7] Mathieu S, Dubost JJ, Tournadre A, Malochet-Guinamand S, Ristori JM, Soubrier M (2010). Effects of 14 weeks of TNF alpha blockade treatment on lipid profile in ankylosing spondylitis. Joint Bone Spine.

[8] Seriolo B, Paolino S, Sulli A, Fasciolo D, Cutolo M (2006). Effects of anti-TNF-alpha treatment on lipid profile in patients with active rheumatoid arthritis. Ann N Y Acad Sci.

[9] Popa C, van den Hoogen FH, Radstake TR, Netea MG, Eijsbouts AE, den Heijer M, van der Meer JW, van Riel PL, Stalenhoef AF, Barrera P (2007). Modulation of lipoprotein plasma concentrations during long-term anti-TNF therapy in patients with active rheumatoid arthritis. Ann Rheum Dis.

[10] Allanore Y, Kahan A, Sellam J, Ekindjian OG, Borderie D (2006). Effects of repeated infliximab therapy on serum lipid profile in patients with refractory rheumatoid arthritis. Clin Chim Acta.

[11] Tam LS, Tomlinson B, Chu TT, Li TK, Li EK (2007). Impact of TNF inhibition on insulin resistance and lipids levels in patients with rheumatoid arthritis. Clin Rheumatol.

[12] Castro KR, Aikawa NE, Saad CG, Moraes JC, Medeiros AC, Mota LM, Silva CA, Bonfá E, Carvalho JF (2011). Infliximab induces increase in triglyceride levels in psoriatic arthritis patients. Clin Dev Immunol.

[13] Peters MJ, Vis M, van Halm VP, Wolbink GJ, Voskuyl AE, Lems WF, Dijkmans BA, Twisk JW, de Koning MH, van de Stadt RJ, Nurmohamed MT (2007). Changes in lipid profile during infliximab and corticosteroid treatment in rheumatoid arthritis. Ann Rheum Dis.

[14] Di Minno MN, Ambrosino P, Peluso R, Di Minno A, Lupoli R, Dentali F, CaRRDs Study Group (2014). Lipid profile changes in patients with rheumatic diseases receiving a treatment with TNF-α blockers: a meta-analysis of prospective studies. Ann Med.

[15] Souto A, Salgado E, Maneiro JR, Mera A, Carmona L, Gómez-Reino JJ (2015). Lipid profile changes in patients with chronic inflammatory arthritis treated with biologic agents and tofacitinib in randomized clinical trials: a systematic review and meta-analysis. Arthritis Rheumatol.

[16] Wijbrandts CA, van Leuven SI, Boom HD, Gerlag DM, Stroes EG, Kastelein JJ, Tak PP (2009). Sustained changes in lipid profile and macrophage migration inhibitory factor levels after anti-tumour necrosis factor therapy in rheumatoid arthritis. Ann Rheum Dis.

[17] Chen DY, Chen YM, Hsieh TY, Hsieh CW, Lin CC, Lan JL (2015). Significant effects of biologic therapy on lipid profiles and insulin resistance in patients with rheumatoid arthritis. Arthritis Res Ther.

[18] Zisman D, Haddad A, Hashoul S, Laor A, Bitterman H, Rosner I, Eder L, Balbir-Gurman A, Mader R, Milman U (2013). Hospitalizations of patients treated with anti-tumor necrosis factor-α agents—a retrospective cohort analysis. J Rheumatol.

[19] Koh KK, Quon MJ, Shin KC, Lim S, Lee Y, Sakuma I, Lee K, Han SH, Shin EK (2012). Significant differential effects of omega-3 fatty acids and fenofibrate in patients with hypertriglyceridemia. Atherosclerosis.

[20] Liao KP, Karlson EW, Hochberg MC, Silman AJ, Smolen JS, Weinblatt ME, Weisman MH (2015). Classification and epidemiology of rheumatoid arthritis. Rheumatology.

[21] Rudwaleit M, Hochberg MC, Silman AJ, Smolen JS, Weinblatt ME, Weisman MH (2015). Classification and epidemiology of spondyloarthritis. Rheumatology.

[22] Armstrong AW, Harskamp CT, Armstrong EJ (2013). Psoriasis and metabolic syndrome: a systematic review and meta-analysis of observational studies. J Am Acad Dermatol.

[23] Dixon WG, Watson KD, Lunt M, Hyrich KL, Silman AJ, Symmons DP, British Society for Rheumatology Biologics Register Control Centre Consortium, British Society for Rheumatology Biologics Register (2007). Reduction in the incidence of myocardial infarction in patients with rheumatoid arthritis who respond to anti-tumor necrosis factor alpha therapy: results from the British Society for Rheumatology Biologics Register. Arthritis Rheum.

[24] Jacobsson LT, Turesson C, Nilsson JA, Petersson IF, Lindqvist E, Saxne T, Geborek P (2007). Treatment with TNF blockers and mortality risk in patients with rheumatoid arthritis. Ann Rheum Dis.

[25] Chen X, Xun K, Chen L, Wang Y (2009). TNF-alpha, a potent lipid metabolism regulator. Cell Biochem Funct.

[26] Palmer SC, Navaneethan SD, Craig JC, Johnson DW, Perkovic V, Hegbrant J, Strippoli GF (2014). HMG CoA reductase inhibitors (statins) for kidney transplant recipients. Cochrane Database Syst Rev.

[27] Sookoian SC, Gonzalez C, Pirola CJ (2005). Meta-analysis on the G-308A tumor necrosis factor alpha gene variant and phenotypes associated with the metabolic syndrome. Obes Res.

[28] Shiau MY, Wu CY, Huang CN, Hu SW, Lin SJ, Chang YH (2003). TNF-alpha polymorphisms and type 2 diabetes mellitus in Taiwanese patients. Tissue Antigens.

[29] Takahashi T, Takahashi K, Yamashina M, Maesawa C, Kajiwara T, Taneichi H, Takebe N, Kaneko Y, Masuda T, Satoh J (2010). Association of the TNF-{alpha}-C-857T polymorphism with resistance to the cholesterol-lowering effect of HMG-CoA reductase inhibitors in type 2 diabetic subjects. Diabetes Care.

